# Bent ab interno needle goniotomy versus gonioscopy-assisted transluminal trabeculotomy in primary open-angle glaucoma: study protocol of a randomized clinical trial

**DOI:** 10.1186/s13063-024-08134-5

**Published:** 2024-05-03

**Authors:** Gabriel Ayub, Ticiana De Francesco, Vital Paulino Costa

**Affiliations:** Department of Ophthalmology, University of Campinas, 251 Vital Brazil St., Campinas, São Paulo, 13083-888 Brazil

**Keywords:** Glaucoma, Trabecular meshwork, Goniotomy, Trabeculotomy, Randomized clinical trial

## Abstract

**Background:**

Minimally invasive glaucoma surgery (MIGS) is a new class of surgeries, which combines moderate to high success rates and a high safety profile. Bent Ab interno Needle Goniotomy (BANG) and Gonioscopy-Assisted Transluminal Trabeculotomy (GATT) are two low-cost MIGS procedures that communicate the anterior chamber to Schlemm’s canal. Most of the available publications on MIGS are either case series or retrospective studies, with different study protocols. The aim of this manuscript is to describe a randomized clinical trial (RCT) protocol to compare the long-term intraocular pressure (IOP) control and the safety of both procedures in eyes with primary open-angle glaucoma.

**Methods:**

This is a parallel, double-arm, single-masked RCT that includes pseudophakic primary open-angle glaucoma (POAG) eyes. After inclusion criteria, medications will be washed out to verify baseline IOP before surgery. Patients will be randomized to BANG or GATT using a sealed envelope. Follow-up visits will be 1, 7, 15, 30, 60, 90, 180, 330 and 360 days after surgery. On PO330, a new medication washout will be done. The main outcome is the IOP reduction following the procedures. Complimentary evaluation of functional and structural parameters, safety, and quality of life will be done after 30, 90, 180, and 360 days.

**Discussion:**

Our study was designed to compare the long-term efficacy and safety of two low-cost MIGS. Most of the published studies on this subject are case series or retrospective cohorts, with different study protocols, which included different types and severities of glaucomas, combined with cataract extraction. Our study only included mild to moderate POAG eyes, with previous successful cataract extraction. Moreover, it provides a standardized protocol that could be replicated in future studies investigating various types of MIGS. This would allow comparison between different techniques in terms of efficacy, safety, and patients’ quality of life.

**Trial registration:**

Retrospectively registered at the Registro Brasileiro de Ensaios Clínicos (ReBEC) platform RBR-268ms5y. Registered on July 29, 2023.

The study was approved by the Ethics Committee of the University of Campinas, Brazil.

## Background

Glaucoma is the leading cause of irreversible blindness worldwide, affecting over 76 million people nowadays and 40 million more in the years to come [1]. Intraocular pressure (IOP) is the main risk factor related to glaucoma progression [2]. IOP can be controlled through the use of topical medications, laser, or surgery [3–5]. Filtering surgery, such as trabeculectomy, and glaucoma drainage device implantation are effective in reducing IOP, but may lead to severe intra- and postoperative complications [6, 7].

Minimally invasive glaucoma surgery (MIGS) emerges as a new class of surgeries, which may result in adequate IOP reduction, although still inferior to filtering procedures, with a high safety profile [8, 9]. MIGS can be divided into four types depending on the pathway activated by each one: suprachoroidal, subconjunctival, ciliary body, and Schlemm’s canal [8, 9].

Schlemm’s canal MIGS lowers the IOP by reducing the trabecular resistance to aqueous humor outflow through Schlemm’s canal and collector channels [10]. This can be achieved by a trabecular bypass, Schlemm’s canal dilation, or trabeculotomy. Bent Ab interno Needle Goniotomy (BANG) and Gonioscopy-Assisted Transluminal Trabeculotomy (GATT) [11] are two low-cost MIGS procedures that communicate the anterior chamber to Schlemm’s canal.

Most of the available publications on MIGS are either case series or retrospective studies, with different study protocols [11–23]. Moreover, these studies frequently include a variety of types of glaucoma, and cataract extraction is often combined with the MIGS procedure. Finally, none of these studies included the washout of antiglaucoma medications to verify the real IOP effect of the procedure [11–23]. The medication confounding factor prevents an objective interpretation and comparison of the postoperative results. The aim of this manuscript is to describe a randomized clinical trial (RCT) protocol to compare the long-term IOP-lowering effect and adverse effect profile of BANG and GATT in eyes with primary open-angle glaucoma.

## Methods

### Setting

This study is an ongoing parallel double-arm, single-masked RCT that includes pseudophakic primary open-angle glaucoma (POAG) eyes. The study is coordinated by the University of Campinas, São Paulo, Brazil, and patients are recruited at two centers in the country (Hospital de Clínicas da Unicamp, Campinas, São Paulo, Brazil, and Hospital Regional de Divinolândia, Divinolândia, São Paulo, Brazil). Patients’ eyes will be randomized to BANG or GATT by a sealed envelope at a 1:1 ratio. The group allocation is masked for the patient.

The primary outcome is the IOP reduction from baseline. Secondary outcomes are a reduction from baseline of medication use, adverse effects after the intervention, and functional, structural, and quality of life evaluation. Patients will be followed for at least 12 months. This article followed the SPIRIT guidelines for its elaboration [24].

### Inclusion and exclusion criteria

The following inclusion criteria are listed: (1) eyes with POAG, defined as an open angle on gonioscopy and structural loss detected by optic coherence tomography (OCT) and/or functional loss observed with visual field examination, (2) age between 40 and 80 years, (3) visual field mean deviation ≥  − 12 dB, (4) retinal nerve fiber layer thickness (RNFLT) ≥ 60 µm, (5) previous uneventful cataract surgery with intraocular lens implantation, (6) best corrected visual acuity (BCVA) ≥ 0.1 at the Snellen chart (LogMAR = 1), (7) IOP ≥ 18 mmHg measured in two occasions with the Goldmann applanation tonometer under a maximum of 3 IOP-lowering medications, and (8) IOP between 20 and 36 mmHg after medication washout.

Exclusion criteria are defined as: (1) secondary open-angle glaucoma (uveitis, corticoid-induced, pseudoexfoliation, pigmentary and juvenile), (2) primary or secondary angle-closure glaucomas, (3) phakic eyes or cataract surgery performed less than 30 days before inclusion, (4) previous glaucoma surgery (other MIGS procedures, glaucoma drainage devices, or cyclophotocoagulation), (5) previous vitreoretinal surgery, and (6) severe corneal opacity.

### Participant recruitment, initial procedures, and randomization

**Fig. 1:**
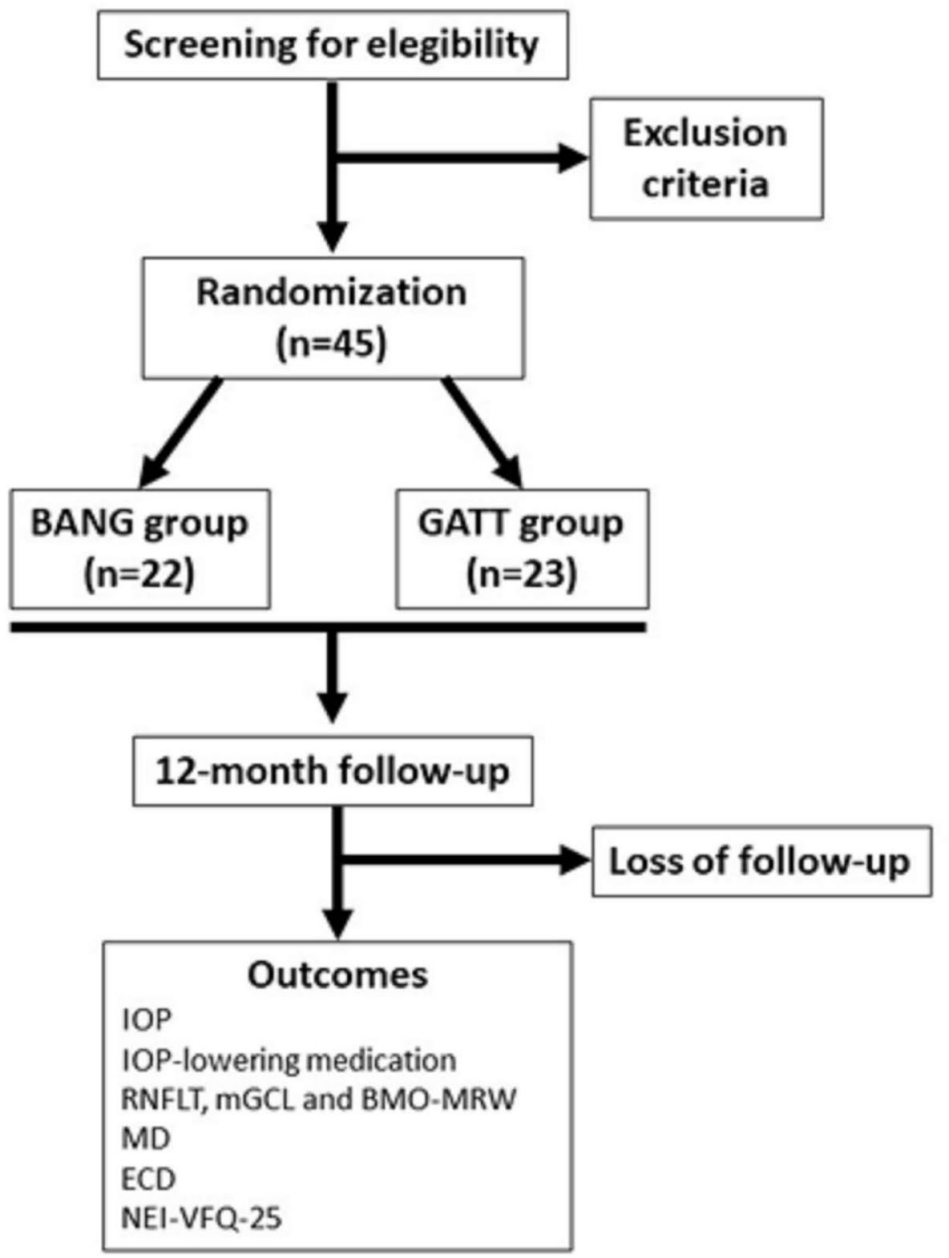
Study flow chart. BANG, bent Ab interno needle goniotomy; GATT, gonioscopy-assisted transluminal trabeculotomy; IOP, intraocular pressure; RNFLT, retinal nerve fiber layer thickness; mGCL, macular ganglion cell layer; BMO-MRW, Bruch’s membrane opening – minimum rim width; MD, visual field mean deviation; ECD, endothelial cell density; NEI-VFQ-25, National Eye Institute Visual Field Function Questionnaire 25

Patients will be recruited at two sites in Brazil. Medical personnel in both sites will be informed of inclusion and exclusion criteria and pre-screening patients who could be eligible for the study. Recruitment will be done by one of the three members of the research team on the same day. The first patient was enrolled in March 2022, and the protocol is still in the recruitment phase. Figure 1 summarizes the participants’ follow-up.

At baseline, a complimentary evaluation will be done, including ultrasonic pachymetry and specular microscopy (Tomey EM3000, Tomey Corporation, Japan) to measure central corneal thickness and endothelial cell density, respectively; RNFLT, macular ganglion cell layer and Bruch’s membrane opening — minimum rim width measurements are obtained with OCT (Spectralis OCT, Heidelberg Engineering, Germany) for structural evaluation; 24–2 visual field tests using SITA-Fast (Humphrey Field Analyzer, Carl Zeiss Meditec AG, Germany) for functional evaluation; and quality of life assessment with the Brazilian validated version of the National Eye Institute—Visual Function Questionnaire-25 questionnaire [25].

After meeting all inclusion criteria at recruitment, patients will be invited to participate in the study. All procedures will be fully explained and an informed consent will be obtained by one of the three members of the research team. After that, patients will be asked to interrupt the use of all IOP-lowering medications and return after 30 days to measure the IOP. The IOP measured after medication washout will be considered the baseline IOP.

### Randomization and group allocation

Randomization will be done using an opaque sealed envelope containing the group (BANG or GATT) at a 1:1 ratio. The random allocation sequence will be generated by the operation room supervisor. The envelope will be opened moments before the surgery by the nurse in the operation room, which will enroll and assign participants for intervention. This study is single-masked for the participants. Double-masking is not possible for investigators, since gonioscopy could identify the surgical procedure postoperatively.

### Interventions

All surgeries will be performed by the same experienced surgeon (GA).

**Fig. 2:**
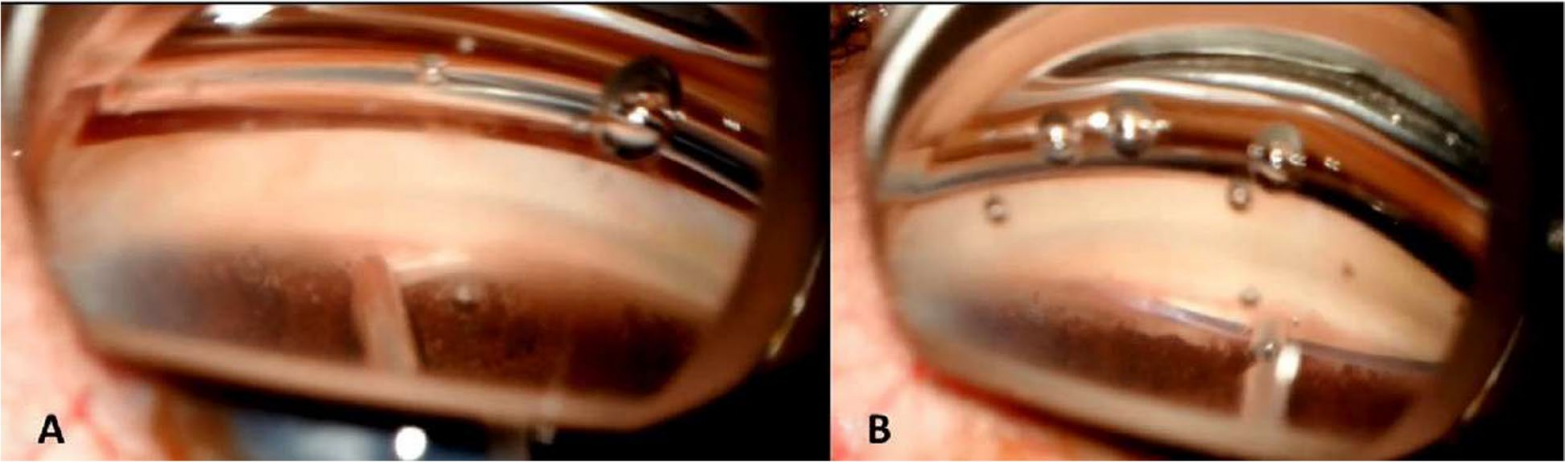
Surgical steps of GATT. **A** A sectorial goniotomy is created with a 25-G needle to expose Schlemm’s canal; **B** the 5–0 Prolene wire is inserted in the canal and progressed 360° using a 23-G microforceps

GATT will be performed as described by Grover et al. [11]. A main incision with a 2.75-mm blade is created temporally. Hydroxypropyl methyl cellulose 4% is injected into the anterior chamber. An auxiliary incision with the 15° blade is made at the supero-temporal of infero-temporal quadrant of the eye. The head of the patient and the microscope are then tilted 45° and 35°, respectively. A surgical sterile gonio lens is used to visualize the nasal trabecular meshwork. Through the main incision, a 25-G needle is used to create a goniotomy of 1–2 mm to expose Schlemm’s canal (Fig. 2A). A 5–0 polypropylene suture wire is inserted 360° through the goniotomy in Schlemm’s canal with the use of a 23-G microforceps (Fig. 2B). The distal edge of the wire is then grabbed by the microforceps, while the proximal edge is pulled, creating a 360° goniotomy. The polypropylene wire is taken out of the eye and the viscoelastic is aspirated by a bimanual irrigation/aspiration device. The incisions are hydrated with a balanced salted solution and sealed.

**Fig. 3:**
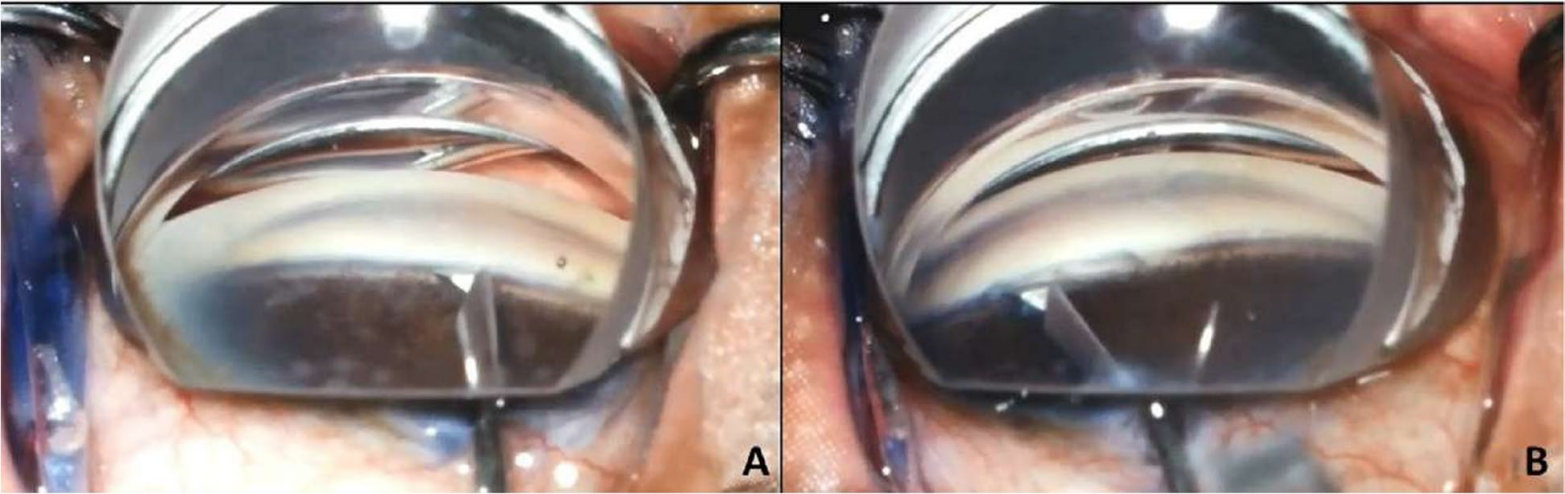
Surgical steps of BANG. **A** A 25-G angled needle tip is inserted in the eye; **B** the trabecular meshwork is excised in the superonasal, nasal, and inferonasal sectors, completing 90° of treatment

BANG will be performed as described by Seibold et al. [26]. A 2.75-mm temporal main incision is created and hydroxypropyl methyl cellulose 4% is injected into the anterior chamber. The head of the patient and the microscope are both tilted 45° and 35°, respectively. A surgical gonioscopy lens is used to visualize the nasal trabecular meshwork. A 25-G angled needle tip is inserted in the eye (Fig. 3A), and a 90° goniotomy is performed in the superonasal, nasal, and inferonasal quadrants (Fig. 3B). Viscoelastic is removed from the eye with the use of a bimanual irrigation/aspiration device. The incisions are hydrated with a balanced salted solution and sealed.

After surgery, a fixed combination of topical dexamethasone 1% s and moxifloxacin 5% drop every 4 h will be prescribed for the first week. After this period, the use of antibiotics will be interrupted, whereas steroids will be tapered during the first postoperative month.

If the patient has both eyes eligible for the study, a different procedure will be performed in each eye. The eye with the highest IOP will be operated first, and the second eye will be operated within a week.

### Follow-up

Patients will be followed 1, 7, 15, 30, 60, 90, 180, 330, and 360 days after surgery (Table 1). In all visits, BCVA and IOP will be measured. Additional visits may be scheduled at the surgeon’s discretion. If in two consecutive visits, the IOP is higher than 18 mmHg, a topical carbonic anhydrase inhibitor will be introduced; if IOP remains above 18 mmHg, the introduction of a second-class IOP-lowering medication will be considered. At visit 330, a new medication washout will be done, and patients will be instructed to interrupt all medications in the operated eye until visit 360.

**Table 1:** Procedures at each timepoint

	**Enrolment**	**Allocation**	**Post-allocation**						**Close-out**
Timepoint**	*** − t***_***1***_	**0**	***t***_***1***_	***t***_***2***_	***t***_***3***_	***t***_***4***_	***t***_***5***_	***t***_***6***_	***t***_***7***_
Description	*** − 30 days***	**0**	***1DPO***	***7DPO***	***30 DPO***	***90 DPO***	***180 DPO***	***330 DPO***	***360 DPO***
Enrolment									
* Eligibility screen*	X								
* Informed consent*	X								
* Allocation*		X							
Interventions									
* Surgery (GATT)*		X							
* Surgery (BANG)*		X							
Assessments									
* Intraocular pressure*	X		X	X	X	X	X	X	X
* Medication use*	X		X	X	X	X	X	X	X
* Functional, structural, and quality of life evaluation*	X				X	X	X		X
* Adverse effects*			X	X	X	X	X	X	X

*DPO* Day postoperative, *GATT* Gonioscopy-assisted transluminal trabeculotomy, *BANG* Bent ab interno needle goniotomy

At visits 30, 90, 180, and 360, all exams obtained at baseline for structural, functional, and quality of life assessment will be repeated. After completing the study endpoint, patients will continue the regular follow-up at the two glaucoma centers enrolled in this study.

During the follow-up period, the investigators will be in close contact with participants (telephone and/or message) to assess any change in visual status. If any change is referred by the participant, a visit will be scheduled for re-evaluation.

Participants will be allowed to withdraw from the study at any time without justification. If possible, patients will be asked to attend for a visit 12 months after surgical intervention to evaluate IOP. Patients who decide to withdraw from the study will be allowed to continue the regular follow-up at the Glaucoma Sector of the recruiting centers without any prejudice.

### Monitoring

The study team is composed of the principal investigator (GA), who supervises the trial and patient management, and study physicians (GA, TDF, and VPC), who identify possible participants. The team will meet monthly to discuss recruitment progress and observe results and harms.

A data monitoring committee was not required during the ethics appreciation process. The study involves two minimally invasive surgical techniques, with literature reporting low rates of adverse effects, and a data monitoring committee would add little value to the safety of the study.

The Ethics committee of the coordinating center will be responsible for auditing the trial conduct periodically. Participants who suffer any harms related to the interventions of the study will be referenced for treatment.

### Confidentiality

Personal data will be collected on paper and electronic forms. All patients will receive an identification number at recruitment.

All paper registries will be kept confidential and stored in a locked box accessed only by the research members. Personal identifiers will be kept available for investigators before the study is completed to ensure cross-validation of participant identity.

Personal identifiers and identification number lists will be safeguarded by the principal investigator (GA) after completion of the study.

### Sample size calculation

A mean difference of 3 mmHg and a standard deviation of 5 mmHg were considered relevant. Considering a statistical power of 80% and a 95% significance, the sample size was calculated as 45 eyes total.

The values considered for this calculation were obtained based on the results of previous goniotomy-like studies [11–23]. Since there were no studies designed to evaluate BANG, the results of studies evaluating the Kahook Dual Blade (KDB) (New World Medical, Rancho Cucamonga, USA) were considered.

### Statistical analysis plan

The normality of the data will be assessed by the Shapiro–Wilk test. Comparison of longitudinal changes in IOP or the number of medications within a group will be done by Student’s paired *t*-test or the Wilcoxon rank sum test depending on the distribution of the data. The comparison of the above-mentioned parameters between the groups will be done by a non-paired Student’s *t-*test or the Mann–Whitney-*U* test depending on the distribution of the data. Categorical variables will be compared using the chi-square or the Fisher exact test.

A Kaplan–Meier survival curve will also be used to compare the success rates of both procedures. Complete success is defined as an IOP reduction ≥ 20% from baseline under no antiglaucoma medication at the final 12-month visit. Qualified success is defined as an IOP reduction ≥ 20% from baseline with the use of medication, but no increase in medication compared to the recruitment visit. Failure is defined as an IOP reduction < 20% from baseline, the need of additional glaucoma surgery to control IOP, IOP < 5 mmHg with the evidence of hypotony maculopathy, or persistent BCVA equal or lower to light perception. The log-rank test will be used to compare the survival curves of both procedures. *P* values < 0.05 will be considered statistically significant.

### Dissemination plans

The results of the study will be presented in ophthalmology meetings and submitted to indexed scientific journals. Authorship eligibility will follow the ICMJE guidelines [27].

## Discussion

### Strengths

Our study provides a standardized protocol that could be used for future studies on MIGS. The strengths of our protocol rely on its single-masked and randomized design. Although several studies on MIGS and goniotomy have been published [11–23], this is, to the best of our knowledge, the first RCT that directly compares those two different standalone trabecular MIGS.

The design of our protocol presents several advantages when compared to the previously published studies evaluating goniotomy-like procedures [11–23]. First, the nature of an RCT reduces the selection bias observed in retrospective studies; moreover, our study will only include patients with previous successful cataract surgery. Phacoemulsification alone is known to promote 13% and 12% reductions in IOP and the number of medications in POAG eyes, respectively [28]. Since previous studies [11–23] mixed standalone and combined procedures, it was not possible to determine the real effectiveness of MIGS on the reduction of IOP and medications. Also, glaucomas other than POAG will not be included in our study. Although some other types of glaucoma are classified as open angle (pigmentary, pseudoexfoliative, corticoid-induced), their pathophysiology is different and the results of surgical procedures may also vary. The inclusion of mild to moderate glaucoma allows MIGS that aim at Schlemm’s canal to achieve target IOP. In severe glaucomas, which typically require low target IOPs, the trabecular meshwork biomechanical pump for aqueous humor outflow is compromised, which may also lead to Schlemm’s canal and collector channels collapse [10], which could influence the success of the goniotomy. Finally, the medication washout before the procedure and at the end of the follow-up will allow to verify the real efficacy of the procedures without the influence of compliance to medical treatment and without the different effects of IOP-lowering medications.

Although our study was designed to compare two goniotomy-like procedures, its design can be expanded to compare other MIGS techniques. The protocol provides a well-established 1-year postoperative follow-up, which is also verified in major glaucoma trials [29–31]. Moreover, it is not limited to IOP control and investigates other parameters, such as functional and structural evaluation, which are important to define glaucoma progression [2], safety (ECD), and patients’ quality of life.

One major barrier for the wider application of MIGS in ophthalmology is the costs involved [32, 33]. Some of the MIGS aiming at Schlemm’s canal are costly, including the Trabectome (Neomedix Inc., Tustin, USA) [26], KDB [34], Hydrus (Ivantis, Inc, Irvine, USA) [35], and iStent (Glaukos Corporation, Laguna Hills, USA) [36]. This limits the utilization of such techniques in developing countries. On the other hand, BANG and GATT are low-cost MIGS. Both techniques require a surgical gonio lens and an additional suture wire or needle, which are low cost [32, 33]. In Brazil, the average cost of a 5–0 Prolene wire used in GATT is around USD 3.00, while a 25-G needle used in BANG costs around USD 0.02. On the other hand, KDB has an estimated cost of USD 400, while iStent, USD 1000. At the time, there are no prospective studies comparing BANG to other MIGS or traditional glaucoma surgeries. Because of the low cost of both procedures, the results of this study could serve as a basis for ophthalmologists of developing countries to choose the type of goniotomy-like procedure they prefer.

### Limitations

Our study has some limitations. Although the sample size was calculated for an 80% statistical power, it is considered a small sample size (*n* = 45 eyes). Also, procedures will be done by a single surgeon, and thus the results cannot be generalized. Moreover, the follow-up will not be masked for the investigator, since gonioscopy would identify the procedure used in that patient.

In summary, our study provides a standardized protocol that could be replicated in future studies investigating various types of MIGS. This would allow comparison between different techniques in terms of efficacy, safety, and patients’ quality of life.

## Trial status

This is the third version of the protocol. The original study was approved in December 2021. The first amendment was approved in February 2022 (inclusion of Hospital Regional de Divinolândia as a participant center) and the second in July 2022 (inclusion of Ticiana de Francesco in the study team).

The trial is in the recruitment phase. Recruitment started in March 2022 and is expected to be completed in March 2024.

## Data Availability

The datasets used and/or analyzed during the current study are available from the corresponding author on reasonable request.

## Abbreviations

MIGS: Minimally invasive glaucoma surgery
BANG: Bent ab interno needle goniotomy
GATT: Gonioscopy-assisted transluminal trabeculotomy
RCT: Randomized clinical trial
IOP: Intraocular pressure
POAG: Primary open-angle glaucoma
ReBEC: Registro Brasileiro de Ensaios Clínicos
OCT: Optic coherence tomography
BCVA: Best corrected visual acuity
RNFLT: Retinal nerve fiber layer thickness
KDB: Kahook Dual Blade
